# Do we need a strong captain to navigate the COVID-19 pandemic? Social identification, conspiracy theory beliefs, and the wish for a strong leader

**DOI:** 10.3389/fpsyg.2023.1100519

**Published:** 2023-02-08

**Authors:** Silvia Moscatelli, Anna Rita Graziani, Lucia Botindari, Stefano Ciaffoni, Michela Menegatti

**Affiliations:** ^1^Department of Psychology, Alma Mater Studiorum University of Bologna, Bologna, Italy; ^2^Department of Communication and Economics, University of Modena and Reggio Emilia, Reggio Emilia, Italy; ^3^SAIS Europe, Johns Hopkins University, Bologna, Italy

**Keywords:** social identification, conspiracy theories belief, COVID-19, wish for a strong leader, trust

## Abstract

**Introduction:**

In times of great uncertainty and hardship, calls for a strong leader tend to arise. The present study delved into this issue by examining possible sociopsychological antecedents of the wish for a strong leader during the COVID-19 crisis.

**Methods:**

We examined the role of social identification, belief in conspiracy theories related to COVID-19, and trust in various relevant social actors in a sample of 350 Italian citizens.

**Results:**

Structural equation modeling analyses showed that identification with Italians was related to a lower wish for a strong leader through the mediation of trust. Identification with Europeans had a direct and negative association with the wish for a strong leader. Finally, greater endorsement of conspiracy beliefs was related to a greater wish for a strong leader, directly and through diminished trust.

**Discussion:**

These findings suggest that belief in conspiracy theories might drive individuals to depart from democratic principles, whereas relying on meaningful social identities can effectively contrast possible authoritarian turns brought about by a global societal crisis, such as the coronavirus outbreak.

## Introduction

The year 2020 undoubtedly left a mark on human history around the world. As the number of infections due to the novel coronavirus, SARS-CoV-2 (COVID-19), increased around the entire globe, so did the numbers of hospitalizations and deceased (e.g., Phelan et al., 2020; WHO, 2020). To prevent further diffusion of the virus, most countries adopted containment and preventive measures that restricted personal freedom, such as national lockdowns, mandatory use of face masks, and social distancing. At the same time, the economic crisis resulting from these restrictions threatened the jobs, wages, and financial security of millions of citizens (e.g., Pak et al., 2020).

Together, these conditions can pose a serious threat to democracy and create an ideal breeding ground for rising calls for a strong leader—that is, a leader who makes firm decisions and is willing to overcome societal difficulties by any means necessary, even if those means are in contradiction to democratic values (Sprong et al., 2019). Political commentators noted that in several countries (e.g., Poland, Hungary, Azerbaijan), the appeal to contain the pandemic served to justify the repression of political opposition or the excessive granting of power to autocratic leaders with the certainty of public support (e.g., Applebaum, 2020; Coynash, 2020; Kurlantzick, 2020; see also Tourish, 2020). In line with these observations, there is some evidence that conditions that foster perceived insecurity and threat – just like the COVID-19 pandemic does – might result in people’s higher levels of authoritarian attitudes and support for anti-democratic systems of government (e.g., Andersen, 2012; Roccato et al., 2020). Moreover, feeling uncertain about one’s world and how one should behave and others will behave increases the preference for strong, “extreme” leaders who have an autocratic leadership style (Hogg and Adelman, 2013).

The present study analyzed possible psychosocial antecedents that buffered or increased the wish for a strong leader during the COVID-19 crisis. First, we drew from research in the social identity framework (Tajfel and Turner, 1979), which emphasized that people cope with crises more effectively if they can rely on a stronger sense of belonging and connectedness to significant groups (Jetten et al., 2020; Moscatelli et al., 2021). Accordingly, we examined whether Italians’ increasing levels of identification with two significant entities involved in facing the pandemic – namely, their nation and the superordinate entity of the European Union (EU) – were related to a lower wish for a strong leader. We also tested whether these relations were accounted for by increased trust in relevant social actors who, through their efforts and behavior, worked to halt the spread of COVID-19 infections (Paolini et al., 2020; Moscatelli et al., 2021; Pagliaro et al., 2021).

Second, we analyzed the role of belief in conspiracy theories, that is, theories that offer explanations of the origins of relevant social events in terms of secret plots by powerful and malevolent actors (for a review, see Douglas et al., 2019). Since pre-pandemic studies underlined that conspiracy theories might foster the support for populism and political extremism (e.g., Fekete, 2012; Vegetti and Littvay, 2021), we tested whether endorsing beliefs in conspiracy theories concerning the pandemic was related to a greater wish for a strong leader through reduced trust.

### Social identification and trust during the COVID-19 crisis

According to social identity theory, belonging to meaningful groups can help individuals deal with social crises (Jetten et al., 2020). Self-defining as a member of a “we” fulfills individuals’ needs for a sense of belonging and meaning (Baumeister and Leary, 1995), and helps them reduce feelings of uncertainty by providing relatively consensual indications about how “prototypical” members should think, feel and behave (Hogg, 2000).

Interestingly for the present study, the more individuals identify with the groups they belong to, the more they trust fellow group members (de Cremer, 2002; La Barbera, 2012; Moscatelli et al., 2014). This might be explained by considering that the awareness of shared category membership fuels a sort of presumptive trust, not based on personal information, which leads to the attribution of positive characteristics to other ingroup members (Brewer, 1996; Kramer, 1999). Social identification also relates to stronger trust in the leader of the group to the extent to which the leader is seen as representative (“prototypical”) of the shared social identity (Giessner and van Knippenberg, 2008; Haslam et al., 2011). Especially during periods of crisis, an identification-based trust may be particularly important as it allows citizens to be confident that their interests will be protected and, as such, facilitates confidence in the leader to emerge (Seijts et al., 2021).

As underlined by the notion of groups as “social cures” (Jetten et al., 2017), social identity provides individuals with a sense of personal control over their fate and collective efficacy. Indeed, stronger group identification is associated with better physical and mental health (Wakefield et al., 2017; Haslam et al., 2018; McNamara et al., 2021), lower risk of depression and stress (Sani et al., 2015), and better coping with psychological and physical difficulties (Jetten et al., 2017). Indeed, studies conducted during the COVID-19 crisis showed that group identification was positively associated with adherence to norms related to infection-reducing behaviors (Graziani et al., 2022; Simić et al., 2022), higher well-being (Paolini et al., 2020; Bowe et al., 2021), and greater perception of support (Stevenson et al., 2021). Moreover, social identification can help keep up morale and optimism during social crises. Building upon the social identity approach, Moscatelli et al. (2021) examined the relationships among Italians’ identification with their country, identification with the European Union (EU), trust in some institutions with a primary role in the management of the pandemic (i.e., the Italian government, the EU, and the scientific community), and the belief that the COVID-19 crisis would eventually result in the improvement of society. Results showed that the more respondents identified with their country and the EU, the more they expected that the COVID-19 pandemic would have some positive repercussions for Italian leaders, the EU, and humankind. Such relationships were partly accounted for by the levels of institutional trust.

### Beliefs in conspiracy theories related to the COVID-19 pandemic

The outbreak of the COVID-19 pandemic was accompanied by a flourishing of conspiracy theories offering explanations for the spreading of the pandemic and its management by governments (e.g., Douglas, 2021; Pellegrini et al., 2021). The endorsement of conspiracy theory beliefs might be driven by epistemic and existential motives (Douglas et al., 2019). The need to believe in conspiracy theories is stronger under conditions of uncertainty (van Prooijen and Jostmann, 2013) or when individuals need to explain especially large-scale events (Leman and Cinnirella, 2013). Believing in conspiracy theory might fulfill the need for agency and control in situations where people do not understand the social world or perceive that the social system in which they live is under threat (Federico et al., 2018).

Widespread conspiracy theories related to the COVID-19 pandemic concerned the idea that the coronavirus was artificially manufactured in laboratories to be used as a bioweapon, despite most evidence leaning towards an explanation in terms of transmission from animal to human (WHO, 2021). Other theories attributed the pandemic to a plot of pharmaceutical companies or claimed that the governments lied about the number of COVID-19-related deaths or the very existence of the pandemic (e.g., Douglas, 2021). For instance, according to a survey conducted in 2020, 38% of Americans and 30% of Italians believed that the COVID-19 death rate had been “deliberately and greatly exaggerated” (Henley and McIntyre, 2020).

Studies showed that individuals who supported COVID-19-related conspiracy theories were less likely to trust experts’ recommendations intended to reduce infection rates and were, therefore, less prone to adhere to prescribed public health measures and to undergo the medical procedures intended to control the contagion (Biddlestone et al., 2020; Juanchich et al., 2021; Enea et al., 2022).

To our knowledge, to date, no studies related conspiracy theories concerning COVID-19 and the wish for a strong leader. However, endorsing conspiracy theory beliefs (unrelated to the pandemic) was found to be associated with lower compliance with social norms, greater acceptance of violence, and stronger intentions to engage in non-normative forms of political action (Jolley and Paterson, 2020; Vegetti and Littvay, 2021). Moreover, individuals high in conspiracy thinking were more likely to embrace populist attitudes (Silva et al., 2017) and use conspiracy theories to discredit political adversaries, thus creating the ideological conditions for political extremism (Fekete, 2012; Douglas et al., 2019).

### Social identification processes, conspiracy theory beliefs, and the wish for a strong leader

Research on the antecedents of the wish for a strong leader highlighted the key role of insecurity and lack of social cohesion. Climates of uncertainty, threat, and economic inequality are associated with reduced trust in government (Rothstein and Uslaner, 2005; Chi and Kwon, 2016), reduced support for democracy (Andersen, 2012; Ceka and Magalhaes, 2020; Jetten et al., 2021), greater appeal of populist parties and radical leaders (Acemoglu and Robinson, 2006; Jay et al., 2019; Russo et al., 2019), and a greater tendency to unquestioningly respect “proper” authorities (Solt, 2012).

Some scholars related the wish for a strong leader to social identity processes. Uncertainty-identity theory (Hogg, 2000) posits that self-uncertainty motivates identification with groups in which leaders are strong, directive, and to varying degrees authoritarian (Hogg and Adelman, 2013). Haslam and Reicher (2007) highlighted that in a group, a failure to develop a sense of shared identity can lead to greater acceptance of authoritarian leadership, which is perceived as better suited to protect the group in the face of threats (Kruglanski et al., 2006). Jetten et al. (2021) argued that economic inequality triggers distrust, enhancing perceived anomie (i.e., breakdown in the social fabric and government; Crimston et al., 2021). In such a situation, leaders who promise to restore order and control are likely to be more appealing than in societies characterized by a less unequal distribution of resources. Moreover, Jay et al. (2019) claimed that economic inequality enhances people’s tendency to cling to national identities and is therefore likely to favor the insurgence of far-right populism. However, such a conceptualization of national identification – in terms of nationalism and focus on the national agendas – should be distinguished from a conceptualization of national identification in terms of attachment and affective commitment to one’s nation (bereft of the belief in its superiority over outgroups; Kende et al., 2019). According to social identity theory, only the latter can work as a driver of social cohesion, trust, and solidarity within the society (Jetten et al., 2017).

Some studies conducted during the COVID-19 pandemic suggested that likewise economic inequality, this crisis might favor an increased request for (or at least greater acceptance of) a strong leader. Amat et al. (2020) reported a widespread willingness to sacrifice individual freedom and sustain technocratic and authoritarian governance in Spain at the beginning of the pandemic. Roccato et al. (2020) found that the exposure to COVID-19 and perceived economic threat due to the pandemic were positively related to preference for anti-democratic political systems in a sample of Italians, regardless of respondents’ pre-pandemic levels of authoritarianism.

In the present study, we were interested in exploring other possible psychosocial factors that can influence the wish for a strong leader during the COVID-19 outbreak, that is, the strength of identification with meaningful groups (one’s nation and the EU) and the endorsement of conspiracy theory beliefs related to the spread of the coronavirus. For these purposes, we conceptualized the strong leader as “someone who aims to overcome difficulties faced by a group or society by any means necessary (including nondemocratic means)” (Sprong et al., 2019, p. 1626). As proved by Sprong et al., the label “strong leader” evokes the idea of a leader who is able to make firm decisions, wishes to change the status quo, and is willing to challenge democratic values and practices and to break the rules if needed to achieve desired outcomes.

## The present study

This research aimed to shed light on the processes that might underlie individuals’ wish for a strong leader during the COVID-19 pandemic. The study was conducted in Italy, the first Western country to be hit by the COVID-19 pandemic, and to impose a strict nationwide lockdown just after China did. Starting on March 9, 2020, Italian schools, services, and non-essential firms were closed for more than 2 months, and Italians were not allowed to leave their homes except for essential food shopping in the proximity of their house. This radical lockdown strategy was just beginning to be loosened in June 2020, when the present study was conducted.

Based on the social identity framework (Tajfel and Turner, 1979; Haslam and Reicher, 2007), we tested whether the strength of identification with one’s nation and the EU was associated with a lower wish of a strong leader. We also examined whether such relations were accounted for by trust in some relevant social actors involved in the management of the pandemic, which was conceived as the expectation that common people, as well as members of the scientific community and health authorities, would do their best to reduce contagion and fight the pandemic. Finally, we analyzed whether endorsing belief in conspiracy theories related to the origins and spread of COVID-19 was positively related to the wish for a strong leader through reduced trust.

Because individuals on the right end of the political spectrum tend to give more value to authorities (Altemeyer, 1998; Sprong et al., 2019), and political partisanship is related to some dimensions of conspiracy theories (such as the beliefs in the concealment of relevant evidence; Farias and Pilati, 2021), we also considered respondents’ political orientations. What’s more, personal experience with COVID-19 was taken into account (e.g., Roccato et al., 2020).

As mentioned, social identification (in terms of ingroup attachment; Kende et al., 2019) fosters trust within groups, helps group members deal with the adverse psychosocial effects of crises, and enhances their perception of collective efficacy and perceived control over uncertainty (Haslam et al., 2018; Bowe et al., 2021). Accordingly, we expected that national identification would be positively related to trust in the social actors involved in the management of the COVID-19 pandemic (Hypotheses 1a), which in turn should be negatively related to the wish for a strong leader (Hypothesis 1b). We also expected a negative association between identification with Italians and the wish for a strong leader, either directly (Hypothesis 1c) or through the mediation of increased trust (Hypothesis 1d). Similarly, we expected identification with Europeans to be positively related to trust (Hypotheses 2a) and negatively associated with the wish for a strong leader, whether directly (Hypothesis 2b) or through increased trust (Hypothesis 2c).

Conspiracy thinking is rooted in the mistrust of authorities (e.g., Milošević Đorđević et al., 2021) and is associated with the endorsement of authoritarian values and anti-democratic attitudes (Fekete, 2012; Sternisko et al., 2020; Vegetti and Littvay, 2021). Thus, endorsing conspiracy theory beliefs related to the COVID-19 pandemic should be associated with lower trust in the social actors in charge of the crisis management (Hypothesis 3a) and positively associated with a higher wish for a strong leader, whether directly (Hypothesis 3b) and/or through decreased trust (Hypothesis 3c). Since conspiracy theories concerning the spread of COVID-19 were in principle unrelated to the specific group memberships we considered in this study, we advanced no hypotheses concerning the relations between endorsing conspiracy theories and identification with Italians and Europeans.

## Method

### Participants

A total of 415 participants were recruited through social media (Facebook, WhatsApp) and snowball sampling. They were invited to participate in a study on the psychological aspects of the pandemic and completed the questionnaire voluntarily. Of the initial respondents, 10 did not provide consent to participate, six were excluded because they were not of Italian nationality, and 49 failed to complete the questionnaire. Therefore, the final sample included 350 Italian participants (273 women, 77 men; *M*_age_ = 37.64 years, *SD* = 14.35 years, range = 18–72 years). This was in line with *a priori* power analysis specifically designed for mediational effects and performed through an R application that reproduces a Monte Carlo simulation approach (Schoemann et al., 2017). We estimated statistical power for a single-mediator model by setting conservative effect sizes among predictors (i.e., expected correlations of 0.20), the mediator, and the outcome variable (Cohen, 1988). Following Schoemann et al. (2017), we chose a large total number of power analysis replications (5000) and wide coefficient draws per replication (20000). The analysis revealed that the final sample of 350 participants allowed the achievement of a statistical power of 0.85 [CI: 0.84; 0.86].

### Procedure

The project was approved by the Bioethical Committee of the first author’s institution. The data analyzed in this paper were collected between June 3 and 18, 2020. The questionnaire was administered anonymously *via* Qualtrics and included other measures (coping strategies, well-being, and compliance with the distancing rules) not considered in the present paper.^1^ Completing the entire questionnaire required approximately 20 min. After providing consent to participate, participants completed measures of identification with Italians and Europeans, belief in conspiracy theories, trust in social actors, and wish for a strong leader. They were then asked about their personal experience with COVID-19 infection and filled in a measure of political orientation. Finally, participants provided demographic information (i.e., gender, age, nationality).

### Measures

Responses for all measures except trust and political orientation were provided on Likert scales ranging from 1 (*strongly disagree*) to 7 (*strongly agree*). The measures of identification with Italians and Europeans each contained three items tapping the sense of belonging to the relevant group (e.g., “I have a sense of belonging to Italians/Europeans”; adapted from Sani et al., 2015). Cronbach’s alphas were 0.81 for identification with Italians and 0.92 for identification with Europeans.

Belief in conspiracy theories was measured using three *ad-hoc* items (“The coronavirus has been created in a laboratory and for some reason escaped scientists’ control;” “The governments of some countries had long been aware of COVID-19 before the spread of the pandemic and preferred to keep quiet;” “Some independent scientists and physicians have found treatment for the coronavirus infections, but their discoveries have been kept secret to enrich drug companies”; α = 0.79).

To measure trust in social actors, participants were asked to rate the extent to which they trusted and believed that the following groups were doing their best to reduce the contagion and fight the pandemic (1 = *not at all* to 5 = *a lot*): health authorities, the scientific community, and other human beings (α = 0.66). The wish for a strong leader was measured using three items adapted from Sprong et al. (2019) referring to the country’s leadership during the pandemic (e.g., “We need strong leadership in order to make our country deal with the COVID-19 pandemic”; α = 0.93).

Two items detected personal experience with COVID-19: “Have you contracted COVID-19?,” “Has a member of your family or a close friend of yours contracted COVID-19?.” Thirty-three participants (9.4%) reported that they had contracted COVID-19 themselves, and 87 (24.9%) reported that a member of their family or a close friend had contracted the virus. Responses to these two questions were collapsed to create the variable “personal experience with COVID-19.” Overall, 107 (30.1%) respondents reported having had personal experience with COVID-19.

Political orientation was measured by asking participants to report their political self-placement on an 11-point left–right continuum (0 = *completely left*, 10 = *completely right*).

## Results

### Preliminary analyses

Table 1 shows the mean values of all measures and the correlations among the model variables. A series of preliminary *t*-tests revealed that participants who had personal experience with COVID-19 showed lower identification with Italians (*M* = 4.55, *SD* = 1.12) than those who had no such personal experience (*M* = 4.81, *SD* = 1.07), *t*(348) = 2.08, *p* = 0.038, *d* = 0.024. There were no other significant differences due to personal experience with COVID-19 (*p*-values > 0.121). There were no significant differences between male and female participants (*p*-values > 0.125).

**Table 1:** Means (standard deviations) of the model variables and correlation matrix.

Measures	*M* (*SD*)	2.	3.	4.	5.	6.
1. Identification with Italians	4.73 (1.09)	0.41***	−0.03	0.27***	−0.06	−0.08
2. Identification with Europeans	4.43 (1.43)	1	−0.33***	0.30***	−0.34***	−0.42***
3. Belief in conspiracy theories	3.22 (1.45)		1	−0.30***	0.38***	0.35***
4. Trust	3.15 (1.63)			1	−0.30***	−0.23***
5. Wish for a strong leader	3.80 (1.73)				1	0.35***
6. Political orientation	3.71 (2.05)					1

Identification with Italians and with Europeans, belief in conspiracy theories, and wish for a strong leader were measured on 7-point scales. Trust was measured on a 5-point scale. Political orientation was measured on a 11-point scale (0 = completely left, 10 = completely right).

*** *p* < 0.001.

### Mediation model

To test the hypotheses, we estimated a model in which the two identification measures and the measure of belief in conspiracy theories were modeled as predictors, trust in social actors was included as the mediator, and the wish for a strong leader was inserted as the outcome variable. The model estimated correlations between predictors. All the above variables were latent variables, with items used as indicators. Political orientation and experience with COVID-19 were included as covariates. To adjust for measurement error, SEM with latent variables (Bollen, 1989) was performed using Mplus version 8.3 (Muthén and Muthén, 2019).

Model parameters were estimated using the maximum likelihood (ML) method. To test for mediation, we calculated bootstrap (5,000 resamples) estimates of indirect effects and bootstrapping bias-corrected confidence intervals (CIs). Mardia’s tests for multivariate skewness (MS) and multivariate kurtosis (MK) run to check for multivariate normality were significant, *MS* = 3.00, *p* < 0.001 and *MK* = 53.21, *p* < 0.001, thus rejecting the multivariate normality assumption (Bentler, 2006). Using bootstrap estimation is recommended in cases of multivariate nonnormality and provides reliable results (Lai, 2018). We also re-run the model using the MRL method, robust to deviations from normality. The results of this additional analysis were comparable with those obtained with the bootstrap and are reported in Supplementary Table S1.

To examine model fit, we relied on the following indices (Schumacker and Lomax, 2010): comparative fit index (CFI) and Tucker–Lewis index (TLI), both of which should exceed 0.90 to be considered acceptable, and root mean square error of approximation (RMSEA) and standardized root mean square residual (SRMR), both of which should be less than 0.08 (Hu and Bentler, 1999). First, we estimated the measurement model. The fit indexes were acceptable, CFI = 0.960, TLI = 0.947, RMSEA = 0.067, SRMR = 0.045. The factor loadings for the items of the latent variables are reported in Supplementary Table S2.

**Figure 1:**
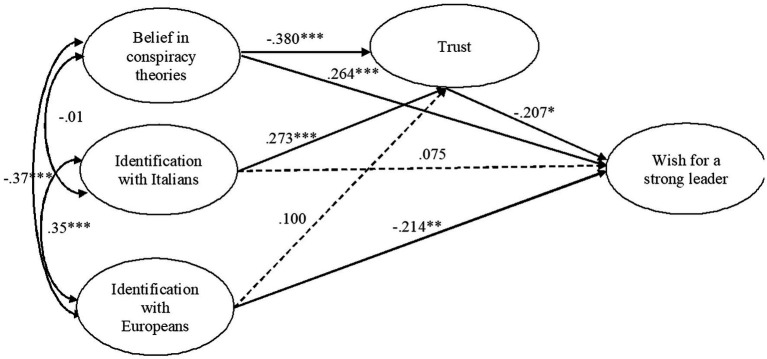
Standardized solution of the model testing the relations among identification with Italians and Europeans, belief in conspiracy theories, trust, and the wish for a strong leader.

The fit indexes of the mediation model were acceptable (CFI = 0.960; TLI = 0.946; RMSEA = 0.061; SRMR = 0.042). Table 2 reports the estimates for direct and indirect effects and the CIs of the main model. Significant direct links are also shown in Figure 1.

**Table 2:** Standardized direct and indirect effects of the SEM.

	Trust in social actors *ß* (SE) [95% CI]	Wish for a strong leader *ß* (SE) [95% CI]
Direct effects		
Identification with Italians	0.273*** (0.074)[0.123, 0.412]	0.075 (0.066)[−0.061, 0.202]
Identification with Europeans	0.100 (0.077)[−0.054, 0.254]	−0.214** (0.066)[−0.339, −0.078]
Belief in conspiracy theories	−0.340*** (0.074)[−0.523, −0.234]	0.264*** (0.068)[0.130, 0.396]
Trust		−0.207** (0.082)[−0.368, −0.045]
Indirect effects		
Identification with Italians ➔ Trust ➔		−0.057* (0.028)[−0.121, −0.010]
Identification with Europeans ➔ Trust ➔		−0.021 (0.020)[−0.070, 0.009]
Beliefs in conspiracy theories ➔ Trust ➔		0.079** (0.034)[0.017, 0.150]
Covariate variables		
Political orientation	−0.097 (0.074)[−0.243, 0.045]	0.156* (0.063)[0.026, 0.272]
Experience with COVID-19	−0.022 (0.084)[−0.186, 0.148]	−0.041 (0.068)[−0.171, 0.095]

* *p* < 0.05;

** *p* < 0.01;

*** *p* < 0.001.

As expected, identification with Italians was significantly related to the proposed mediator (i.e., trust; Hypothesis 1a), which in turn was negatively associated with the wish for a strong leader (Hypothesis 1b). Whereas the direct link between the predictor and the wish for a strong leader was not significant (Hypothesis 1c), the indirect link through trust was significant, supporting Hypothesis 1d.

Unlike what we expected, identification with Europeans was not significantly associated with trust (Hypothesis 2a). Supporting Hypothesis 2b, identification with Europeans had a direct and negative significant association with the wish for a strong leader. The indirect link through trust was not significant (Hypothesis 2c).

Belief in conspiracy theories was negatively related to trust (Hypothesis 3a). Supporting the expectations, the analysis revealed a significant direct association between belief in conspiracy theories and the wish for a strong leader (Hypothesis 3b) and a significant indirect link through trust (Hypothesis 3c).

Additionally, the analysis revealed some significant associations between political orientation and the main variables. The more respondents were right-wing oriented, the less they identified with Europeans, *β* = −0.287, *SE* = 0.062, *p* < 0.001, 95% CI [−0.407, −0.160]. Moreover, the more respondents were right-wing oriented, the more they endorsed conspiracy theory beliefs, *β* = 0.178, *SE* = 0.073, *p* = 0.014, 95% CI [0.039, 0.319] and reported a wish for a strong leader, *β* = 0.156, *SE* = 0.063, *p* = 0.014, 95% CI [0.026, 0.272]. No significant relationships between the experience with COVID-19 and the model’s main variables emerged (*p*-values >0.153).

Since scholars recently claimed that conducting analyses both with and without control variables can be valuable for statistical validity (Sturman et al., 2022), we re-run the analyses without including political orientation and personal experience of COVID-19 as covariates. The fit of this model was acceptable (CFI = 0.960; TLI = 0.947; RMSEA = 0.067; SRMR = 0.045). Results remained unchanged when the covariates were removed, supporting that the covariates did not alter the relationships between the main variables. The direct and indirect effects are reported in Supplementary Table S2.

## Discussion

The present study aimed to shed light on some possible antecedents of the wish for a strong leader during the COVID-19 pandemic. To this end, it explored the predictive role of social identification with Italians and Europeans, belief in conspiracy theories related to COVID-19, and trust in social actors. Overall, the findings supported the general expectation that social identification may represent a buffer against the wish for a strong leader, which is instead enhanced by belief in conspiracy theories.

As expected, greater identification with Italians was related to enhanced trust in social actors and, through trust, to a decreased wish for a strong leader. Moreover, the more that respondents thought of themselves as Europeans, the less they felt that a strong leader was needed to deal with the pandemic. However, such a link was not mediated by trust in social actors. Although these findings highlight that the feeling of being part of one’s country and a superordinate entity such as the EU can help counteract the call for a more authoritarian leader, they also suggest that the two social identifications might accomplish this end in different ways.

To interpret these findings, it is important to consider the peculiarities of the situation and the context in which the research took place. In the first months of the COVID-19 crisis, Italians showed a sharp increase in national identification and trust in the national government—a phenomenon that often occurs when a country is under threat and is known as the “rally-round-the-flag” effect (e.g., Falcone et al., 2020). The intense feeling of a shared fate stemming from the awareness of being the first country in the Western world to deal with the COVID-19 outbreak likely strengthened Italians’ sense of intragroup connection and boosted their trust that everyone would do their best to fight the pandemic. This, in turn, seems to counter the perceived necessity for strong leadership.

Whereas this finding seems in line with the “groups as social cures” assumption (e.g., Jetten et al., 2017), we recognize that, in principle, one might have expected national identification to be related to right-wing political orientation as well as to higher levers of wish for a strong leader. In fact, in the Italian political debate, national identity, patriotism, and defense of the national traditions from immigration are key arguments of right-wing parties (Andrew, 2017; Caricati, 2019), which are the ones that emphasize the need for strong authorities (Altemeyer, 1998; Sprong et al., 2019). However, the COVID-19 pandemic has likely increased citizens’ national identification regardless of their political orientation, as revealed by the lack of correlation between the two measures observed in the present study. In our view, the increased importance of one’s own country as a social identity for all Italian citizens (e.g., Cárdenas et al., 2021) might explain why identification with Italians was not significantly associated with political orientation and the wish for a strong leader in the present research.

Identifying with Europeans likely fulfilled a different function than national identification. Italians have traditionally been among the staunchest supporters of European integration, viewing membership in the EU as a path toward societal modernization and as something of a bulwark against domestic economic and political turmoil (Balfour and Robustelli, 2019; Moscatelli et al., 2019). Despite the slight worsening of Italians’ attitudes and feelings toward the EU at the beginning of the COVID-19 outbreak (e.g., Berti, 2020; Fontana, 2020), it seems plausible that even during such a crisis, the key function fulfilled by identification with Europeans was helping Italians to maintain control over political and societal uncertainties in the face of the threat posed by the pandemic. The direct link between European identification and the wish for a strong leader observed in this study might therefore be due to the specific nature of Italians’ ties with the EU. Of course, further research is required to support these conclusions.

Endorsing beliefs in conspiracy theories was – as expected – related to a higher wish for a strong leader, both directly and through the mediation of decreased trust in social actors. Thus, the spread of conspiracy beliefs about COVID-19 is not only likely to reduce individuals’ adherence to restrictive measures and vaccination campaigns (e.g., Biddlestone et al., 2020; Hornsey et al., 2021; Simić et al., 2022) but can even affect their support for democratic principles. A key component of conspiracy narratives about the pandemic is that the state (or the elites who control the state) has purposely hidden the truth from the common people (Douglas, 2021). Such arguments, which erode trust in the state and its institutions, can undermine democracy and support people’s faith in leaders who can take over from the elites accused of deceiving the people regarding the current situation.

We had no hypotheses concerning the relationship between believing in conspiracy theories concerning COVID-19 (which, as measured in this study, did not refer to the possible role of specific countries or groups in the spread of the virus) and social identification. Endorsing conspiracy theory beliefs was not associated with identification with Italians, whereas it was negatively related to identification with Europeans. Such a result suggests that identifying with a superordinate entity such as the EU – which represents an anchor for Italians in the face of national political and economic instability (Balfour and Robustelli, 2019) – might somehow work as a buffer against the allure of “simple” explanations of global crises such as the COVID-19 pandemic. Alternatively, it might as well be that people who tend to endorse conspiracy theory beliefs are more likely to be skeptical of the EU’s authorities and goals. Although our findings do not allow us to disentangle the two possible interpretations, this issue might have relevant implications and should be addressed in future research.

Our study also revealed positive associations between right-wing political orientation, endorsing conspiracy theories, and the wish for a strong leader. Such a finding is in line with the claim that the pandemic has been politicized in some Western countries (including Italy; Salvati et al., 2022), resulting in conservatives and voters of right-wing populist parties endorsing conspiracy theory beliefs to a higher extent than left-oriented voters (e.g., Havey, 2020; Farias and Pilati, 2021; Ruisch et al., 2021). Indeed, since the beginning of the pandemic, there has been a convergence of supporters of conspiracy theories and the extreme political right in countries such as the United States, the United Kingdom, Hungary, and Italy (e.g., Buranyi, 2020; Calvillo et al., 2020; Havey, 2020). Such a relationship between political ideology and the endorsement of conspiracy theory beliefs should be considered contingent on the specific issues and contexts considered in the mentioned studies (e.g., Calvillo et al., 2020): As argued by Sternisko et al. (2020), members of different groups might come to endorse specific conspiracy theories insofar as their contents allow affirming the superiority of one’s group while denigrating outgroups.

### Limitations and future research

This study has some limitations. First, relying on cross-sectional data does not allow us to rule out different inferences concerning the causal direction of the effects. Based on social identity theory (Tajfel and Turner, 1979) and previous evidence on the predictors of people’s responses to COVID-19 (Moscatelli et al., 2021; Simić et al., 2022), we assumed that trust in social actors is founded on and strengthened by intragroup considerations related to one’s experience within social groups. However, one might claim that trust could be considered a general and relatively stable individual expectancy regarding others’ intentions and behavior, which precedes people’s tendency to identify with social groups (e.g., Sorrentino et al., 1995). Experimental or longitudinal designs are necessary to prove our assumption over alternative paths.

Whereas we focused on conspiracy theories related to the origins and spread of COVID-19, conspiracy theories are more complex and multifaceted. Since it has been shown that different narratives can lead to different outcomes (e.g., Hornsey et al., 2021), future studies should consider the specific impacts of different conspiracy theories on believers’ attitudes and behaviors, including the wish for societal change. Concerning our measure of conspiracy theory beliefs, one might also question whether one of the items we employed (“The coronavirus has been created in a laboratory and for some reason escaped scientists’ control”) conveyed the key conspirationist idea that malevolent forces are involved in the origins of the COVID-19. Even though we are relatively confident that respondents understood the item’s intended meaning given the ongoing debates on the origins of the coronavirus when the data collection took place, we recognize that this item was ambiguous and that the “lab-leak” hypothesis even found the support of some scientists (Maxmen and Mallapaty, 2021; see, however, Andersen et al., 2020; WHO, 2021; Worobey, 2021, for critical stances on the lab-leak hypothesis). To be sure that the use of such a specific item did not compromise the overall findings, we ran the mediation analysis excluding it from the measure of conspiracy theory beliefs. The findings (reported in Supplementary Table S3) showed virtually identical patterns to those described in the Results section, with conspiracy theory beliefs being negatively related to trust and, through it, the wish for a strong leader.

We acknowledge that the definition of strong leader used here – adapted from Sprong et al. (2019) and Jetten et al. (2021), among others – does not go into detail about leadership styles, nor does it relate leaders’ behaviors to specific models of leadership. Although the label “strong leader” has the potential to evocate the desired meaning in laypeople (Sprong et al., 2019), different types of leaders (e.g., transformational, authentic, and so on) can adopt the behaviors associated with such a view of strong leader (e.g., taking firm decisions). Future studies might examine more in-depth how citizens come to accept authoritarian changes from otherwise different leaders in times of global crisis and how leaders with different styles are successful in dealing with it (e.g., Tourish, 2020).

Whereas in this study we focused on trust in some of the relevant actors involved in halting the spread of the pandemic, future research could consider the role of trust in the government and the leader in charge, delving into the specific social and political context. In general terms, it seems plausible that endorsing conspiracy beliefs would be associated with lower trust in the government and a higher wish for a strong leader (as found in this study). However, people who endorse conspiracy theories might even come to trust the leader in charge more and wish for a strengthening of their leadership rather than a political change if the leader is seen as close to the conspirationist positions (i.e., if the leader is perceived as prototypical of the conspirationist group; Haslam et al., 2011). For instance, this might be the case of leaders who are skeptical of the scientists’ position, as happened in the US during Donald Trump’s presidency (Chan et al., 2021; Gu et al., 2021). Of course, in such a situation, people’s support for stronger leadership is unlikely to result in a higher effectiveness of the leader in charge in managing the pandemic, especially if the leader endorses conspiracy beliefs and opposes scientific solutions (Chan et al., 2021).

## Conclusion

These findings contribute to the literature on the role of social identification processes in dealing with social crises (Jetten et al., 2017, 2020). Keeping in mind the limitations of a correlational study, our results seem to suggest that identification with one’s country and identification with the EU might contribute to psychological coping in different ways. National identification seems to have more general positive returns on trust in other people, health authorities, and the scientific community. In contrast, identifying as European appears to be more directly related to a lower wish for a strong leader in response to the COVID-19 crisis. Albeit correlational, such findings open new possible avenues for research in the social identity realm and point out the importance of nurturing people’s identification with their own country, trust in the social actors involved in facing social crises, and citizens’ sense of being part of a superordinate common group that cares about the subgroups within it (Gaertner et al., 2016).

These findings also contribute to the knowledge of the correlates of conspiracy theory beliefs. Research has mainly uncovered the costs of conspiracy thinking related to the COVID-19 pandemic in terms of compliance with public health measures (e.g., Biddlestone et al., 2020; Hornsey et al., 2021). This study reveals that the spread of conspiracy theories can also enhance the followers’ acceptance of or support for more authoritarian leaders.

Finally, this study adds to the existing literature on the antecedents of the wish for a strong leader (e.g., Sprong et al., 2019). Jetten et al. (2021) noticed that economic inequality has the potential to increase citizens’ acceptance of a strong leader as well as their endorsement of beliefs in conspiracy theories that provide simple explanations for the causes of economic inequality. Our findings suggest that in situations of crisis, the spread of conspiracy theories might contribute to enhancing the attractiveness of a strong leader by decreasing trust in those who are in charge of managing the crisis. Thus, reducing people’s susceptibility to COVID-19 conspiracy theories is important not only in containing the virus (e.g., Biddlestone et al., 2020; Douglas, 2021) but also in contrasting possible societal turmoil.

Although conspiracy theories are generally quite resistant to disconfirmation (e.g., Lewandowsky et al., 2012), governments should develop interventions to fight the spread of conspiracy theories and increase people’s exposure to scientifically supported information about COVID-19. Since conspiracy theories are often tied to specific social and political identities (e.g., Chayinska and Minescu, 2018; Sternisko et al., 2020), it is crucial that interventions aimed to contrast them start from an accurate examination of the contents of the beliefs endorsed by different groups as key elements of their identities. Interventions should consider how social media covered the COVID-19 pandemic and how the political debates address specific issues (Calvillo et al., 2020; Havey, 2020). Given that people are more susceptible to information coming from valued ingroup sources, a strategy based on “trusted messengers” (Douglas, 2021, p. 272), rather than scientists or other sources likely to be perceived as outgroup members, might be efficacious in this area.

In conclusion, this research adds a new component to the understanding of the possible antecedents of the wish for a strong leader, which might represent a step toward an authoritarian turn in society. Whereas belief in conspiracy theories can undermine individuals’ endorsement of democratic values, relying on meaningful social identities can efficaciously contrast the possible anti-democratic temptations brought about by a global societal crisis, such as the COVID-19 outbreak.

## Data availability statement

The datasets presented in this study can be found in online repositories. The names of the repository/repositories and accession number (s) can be found at: https://osf.io/37eub/?view_only=5588d89850b04635b1557cd25e40c006.

## Ethics statement

The studies involving human participants were reviewed and approved by Bioethical Committee of the University of Bologna. The patients/participants provided their written informed consent to participate in this study.

## Author contributions

SM, ARG, and LB conceived and designed the study. All authors collected the data. SM, MM, and SC conducted the statistical analyses. ARG funded the research. SM wrote the first draft. All authors contributed to the article and approved the submitted version.

## Funding

The present work has been carried out thanks to the support of the Fondo di Ateneo per la Ricerca 2022 (FAR; University Research Fund) of the Department of Communication and Economics, University of Modena and Reggio Emilia.

## Conflict of interest

The authors declare that the research was conducted in the absence of any commercial or financial relationships that could be construed as a potential conflict of interest.

## Publisher’s note

All claims expressed in this article are solely those of the authors and do not necessarily represent those of their affiliated organizations, or those of the publisher, the editors and the reviewers. Any product that may be evaluated in this article, or claim that may be made by its manufacturer, is not guaranteed or endorsed by the publisher.

## Footnotes

^1^The present research is part of a larger project in which many other variables were assessed for different purposes. Findings concerning some of these variables (i.e., intentions to use the tracing app and to be vaccinated against COVID-19) were included in Graziani et al. (2022).
