# Supplementary material for: Do we need a strong captain to navigate the COVID-19 pandemic? Social identification, conspiracy theory beliefs, and the wish for a strong leader
Source: Front Psychol. 2023 Feb 8;14:1100519. doi: 10.3389/fpsyg.2023.1100519 (PMC9945967; doi:10.3389/fpsyg.2023.1100519)
Supplement: Supplementary file 1 [file Data_Sheet_1.docx]

Supplementary Material

Table 1S shows the results of the alternative model estimated with MLR, which is a robust estimator when the data do not respect multivariate normality; the results are very similar to those described in the Results section.

Table 2S shows the factor loadings for the items of the latent variables resulting from the measurement model.

Table 3S shows the direct and indirect effects of a model without covariates; the results are very similar to those described in the Results section.

Table 4S shows the direct and indirect effects of the mediational model run with a 2-item measure of belief in conspiracy theory. Since we recognize that one of the items employed in the measure of belief in conspiracy theories did not clearly indicate a malevolent force responsible for the origins and spread of COVID-19 (a key element of conspiracy narratives; Douglas et al., 2019), we run the mediation analyses excluding that item (i.e., “The coronavirus has been created in a laboratory and for some reason escaped scientists’ control”). The results were very similar to those described in the Results section.

*Table 1S*. Standardized direct and indirect effects of the SEM (MLR estimator)

|  | Trust in social actors | Wish for a strong leader |
| --- | --- | --- |
|  | *ß* (SE)  [95% CI] | *ß* (SE)  [95% CI] |
| Direct effects |  |  |
| Identification with Italians | .273*** (.071) | .075 (.065) |
| Identification with Europeans  Belief in conspiracy theories  Trust | .100 (.075)  -.380*** (.071) | -.214** (.065)  .264*** (.066)  -.207** (.079) |
|  |  |  |
| Indirect effects |  |  |
| Identification with Italians 🡪  Trust🡪 |  | -.057* (.026) |
| Identification with Europeans 🡪  Trust🡪 |  | -.021 (.019) |
| Beliefs in conspiracy  theories 🡪 Trust 🡪 |  | .079* (.032) |

*Note*. * *p* < .05; ** *p* < .01; *** *p* < .001.

Model fit indexes: CFI = .958; TLI = .943; RMSEA = .057; SRMR = .042.

*Table 2S*. Factor loadings for the latent variables in the measurement model

| Items | Identification with Italians | Identification with Europeans | Conspiracy theory beliefs | Trust in social actors | Wish for a strong leader |
| --- | --- | --- | --- | --- | --- |
| I have a sense of belonging to Italians | .497 |  |  |  |  |
| Being Italian is important to me | .943 |  |  |  |  |
| I have a lot in common with Italians | .764 |  |  |  |  |
| I have a sense of belonging to Europeans |  | .967 |  |  |  |
| Being European is important to me |  | .954 |  |  |  |
| I have a lot in common with Europeans |  | .751 |  |  |  |
| CTB – Coronavirus created in a lab |  |  | .829 |  |  |
| CTB – Governments were aware long before |  |  | .666 |  |  |
| CTB – Discoveries of independent scientists kept secret |  |  | .764 |  |  |
| Trust in health authorities |  |  |  | .749 |  |
| Trust in the scientific community |  |  |  | .594 |  |
| Trust in human beings |  |  |  | .542 |  |
| We need strong leadership in order to make our country deal with the COVID-19 pandemic |  |  |  |  | .868 |
| Our country needs a strong head of government to overcome the difficulties right now |  |  |  |  | .915 |
| We need a leader able to make firm decisions to make our nation overcome the pandemic |  |  |  |  | .921 |

*Note*. CTB = Conspiracy theory beliefs

*Table 3S*. Standardized direct and indirect effects of the SEM estimating a model without covariates

|  | Trust in social actors | Wish for a strong leader |
| --- | --- | --- |
|  | *ß* (SE)  [95% CI] | *ß* (SE)  [95% CI] |
| Direct effects |  |  |
| Identification with Italians | .271*** (.075)  [.120, .412] | .075 (.066)  [-.059, .203] |
| Identification with Europeans    Belief in conspiracy theories    Trust | .102 (.077)  [-.053, .254]  -.340*** (.074)  [-.523, -.231] | -.214** (.066)  [-.337, -.077]  .263*** (.068)  [.130, .394]  -.210** (.082) |
|  |  | [-.370, -.047] |
| Indirect effects |  |  |
| Identification with Italians 🡪  Trust🡪 |  | -.057* (.028)  [-.121, -.011] |
| Identification with Europeans 🡪  Trust🡪 |  | -.021 (.021)  [-.071, .009] |
| Beliefs in conspiracy  theories 🡪 Trust 🡪 |  | .080* (.034)  [.018, .150] |

*Note*. * *p* < .05; ** *p* < .01; *** *p* < .001.

*Table 4S*. Standardized direct and indirect effects of the SEM (with a 2-items measure of conspiracy theory beliefs)

|  | Trust in social actors | Wish for a strong leader |
| --- | --- | --- |
|  | *ß* (SE)  [95% CI] | *ß* (SE)  [95% CI] |
| Direct effects |  |  |
| Identification with Italians | .271*** (.078)  [.113, .415] | .076 (.067)  [-.059, .207] |
| Identification with Europeans    Belief in conspiracy theories    Trust | .110 (.077)  [-.039, .268]  -.352*** (.078)  [-.501, -.192] | -.210** (.067)  [-.335, -.078]  .261*** (.070)  [.123, .400]  -.218** (.081) |
|  |  | [-.378, -.053] |
| Indirect effects |  |  |
| Identification with Italians 🡪  Trust🡪 |  | -.059* (.029)  [-.125, -.012] |
| Identification with Europeans 🡪  Trust🡪 |  | -.024 (.022)  [-.077, .006] |
| Beliefs in conspiracy  theories 🡪 Trust 🡪 |  | .077** (.032)  [.018, .145] |

*Note*. * *p* < .05; ** *p* < .01; *** *p* < .001.

Model fit indexes: CFI = .958; TLI = .940; RMSEA = .066; SRMR = .043.
